# Cross-Cultural Modification Strategies for Instruments Measuring Health Beliefs About Cancer Screening: Systematic Review

**DOI:** 10.2196/28393

**Published:** 2021-11-18

**Authors:** Fang Lei, Eunice Lee

**Affiliations:** 1 University of California, Los Angeles Los Angeles, CA United States

**Keywords:** cancer screening, health beliefs, instrument modification, strategy, systematic review

## Abstract

**Background:**

Modification is an important process by which to adapt an instrument to be used for another culture. However, it is not fully understood how best to modify an instrument to be used appropriately in another culture.

**Objective:**

This study aims to synthesize the modification strategies used in the cross-cultural adaptation process for instruments measuring health beliefs about cancer screening.

**Methods:**

A systematic review design was used for conducting this study. Keywords including constructs about instrument modification, health belief, and cancer screening were searched in the PubMed, Google Scholar, CINAHL, and PsycINFO databases. Bowling’s checklist was used to evaluate methodological rigor of the included articles. Results were reported using the PRISMA (Preferred Reporting Items for Systematic Reviews and Meta-analyses) approach with a narrative method.

**Results:**

A total of 1312 articles were initially identified in the databases. After removing duplications and assessing titles, abstracts, and texts of the articles, 18 studies met the inclusion criteria for the study. Based on Flaherty’s cultural equivalence model, strategies used in the modification process included rephrasing items and response options to achieve semantic equivalence; changing subjects of items, changing wording of items, adding items, and deleting items to achieve content equivalence; adding subscales and items and deleting subscales and items to achieve criterion equivalence. Solutions used to resolve disagreements in the modification process included consultation with experts or literature search, following the majority, and consultation with the author who developed the scales.

**Conclusions:**

This study provides guidance for researchers who want to modify an instrument to be used in another culture. It can potentially give cross-cultural researchers insight into modification strategies and a better understanding of the modification process in cross-cultural instrument adaptation. More research could be done to help researchers better modify cross-cultural instruments to achieve cultural equivalence.

## Introduction

Cancer is one of the leading causes of death in the world [1]. In 2018, there were 18.1 million new cases and 9.5 million cancer-related deaths globally [1]. By 2040, the number of new cancer cases is expected to rise to 29.5 million and the number of cancer-related deaths is estimated to climb to 16.4 million [1]. An effective tool to reduce deaths from cancer [2], screening helps detect cancer at the early stage and reveal cancer before symptoms appear [3]. For more than a half century, cancer screening has been an essential component to decrease the burden of morbidity and mortality from cancer [4].

Although cancer screening has proven to be an effective way to detect cancer at the early stage, the use of cancer screenings is not optimal among several populations [4]. Previous research showed that the uptake of cancer screening was associated with health belief of cancer screening [5]. Beliefs and attitudes about cancer screening, such as mistrust of cancer screening and the health care system, beliefs toward the cancer screening process or illness, and fatalistic beliefs, are important factors influencing the participation of high-risk populations in cancer screening [6-10]. Among minority ethnic groups, traditional cultural values, health beliefs about concepts of preventive health, fear of cancer screening, belief that cancer screening is unnecessary unless one is ill, misconceptions concerning one’s susceptibility to cancer, and stigmatization may also deter high-risk populations from getting cancer screening [11].

Based on the health belief model, health belief of cancer screening can be measured by 6 constructs, including perceived severity and susceptibility of cancer, perceived benefits and barriers of cancer screening, self-efficacy, and cues to action of cancer screening [12]. Previous researchers have developed several instruments to measure these constructs on health beliefs of cancer screening [13,14]. One of the most widely used scales [15] is Champion’s Health Belief Model Scale, originally developed to measure the health beliefs of US populations toward breast cancer screening [14]. Later it was translated and modified to different language versions to test health beliefs of cancer screening in other countries and cultures (eg, other groups of people who hold similar values and beliefs about health behaviors) [16].

Cross-cultural instrument adaptation includes 2 necessary steps, instrument translation and instrument modification [17]. Adapting an instrument to be used in another culture is not merely translating the instrument to another language. Since cultural backgrounds vary among different population groups, modifying the instrument to meet the cultural equivalence is essential for ensuring the reliability and validity of translated instruments [18]. According to Flaherty et al [19], a 5-stage equivalence should be met to maintain the integrity of the translated instrument: (1) semantic equivalence ensures the meaning of each item remains conceptually and idiomatically the same, (2) content equivalence ensures the content of each item in the instrument has consistent cultural relevance, (3) technical equivalence ensures the methods of data collection (interviews, observation, or self-report) elicit comparable data, (4) criterion equivalence establishes the normative interpretation of the variable, and (5) conceptual equivalence ensures the same theoretical construct is being measured in each culture.

Compared to a newly developed instrument, using various modification methods to adapt an existing instrument to be used in the target population is a cost efficient and time-saving choice [17]. Although reasons for considering modifications in the adaptation process, including missing concepts or dimensions, different meaning of concepts, different interpretation of terms or phrases, different style of responding, and complex or difficult respond options, were reported in a previous study [18], ways to use the modification strategies to adapt the instrument to measure health belief of cancer screening in another population/ethnicity to achieve cultural equivalence were not reported. Synthesizing the modification strategies used in the cross-cultural instrument adaptation process will provide general guidance to help novice researchers gain deeper insight into the instrument modification process.

The purpose of this systematic review was to synthesize the modification strategies used in the cross-cultural instrument adaptation process measuring health belief of cancer screening for another population/ethnicity based on Flaherty’s cultural equivalence model [19], especially focusing on semantic, content, and criterion equivalence. This study will provide guidance for researchers who want to modify an instrument to be used in another culture. The modification strategies synthesized in the findings of this study could be further generalized to the modification process of other instruments.

## Methods

A systematic review design was used for conducting this study. Keywords including constructs about instrument modification, health belief, and cancer screening were searched in the PubMed, Google Scholar, CINAHL, and PsycINFO databases in June 2020. Detailed keywords from each construct included (1) instrument modification: instrument, modify, revise, adapt, adaptation, refinement, refine; (2) health belief: perception, attitude, belief, perspective; and (3) cancer screening: cancer, screening, prevent, prevention. Equivalent index terms with the same meanings were also searched. Inclusion criteria for the articles were (1) peer-reviewed articles, (2) reported instrument modification process about health beliefs toward cancer screening, and (3) published in the English language (which could be read by the authors). The exclusion criteria were (1) informal articles such as commentary, letter to the editor, and conference abstract and (2) constructs from the health belief model not included.

For identifying relevant studies, keywords were applied to search the full text of articles in the databases. Titles and abstracts of the articles were read further to exclude irrelevant studies. Articles in the reference list of selected articles were also searched. Inclusion and exclusion criteria were evaluated during the process. Information on the purpose, sample, setting, methods, results, and discussion of the included articles was extracted and entered into the table of evidence. Bowling’s checklist was used to evaluate methodological rigor of the included articles [20]. The studies’ aims, methods, results, and conclusions were evaluated by assessing the 20 items in Bowling’s checklist. Results in the study were reported using a narrative method following the PRISMA (Preferred Reporting Items for Systematic Reviews and Meta-analyses) guidelines [21] (see Multimedia Appendix 1 for PRISMA checklist).

## Results

### Search Findings

**Figure 1:**
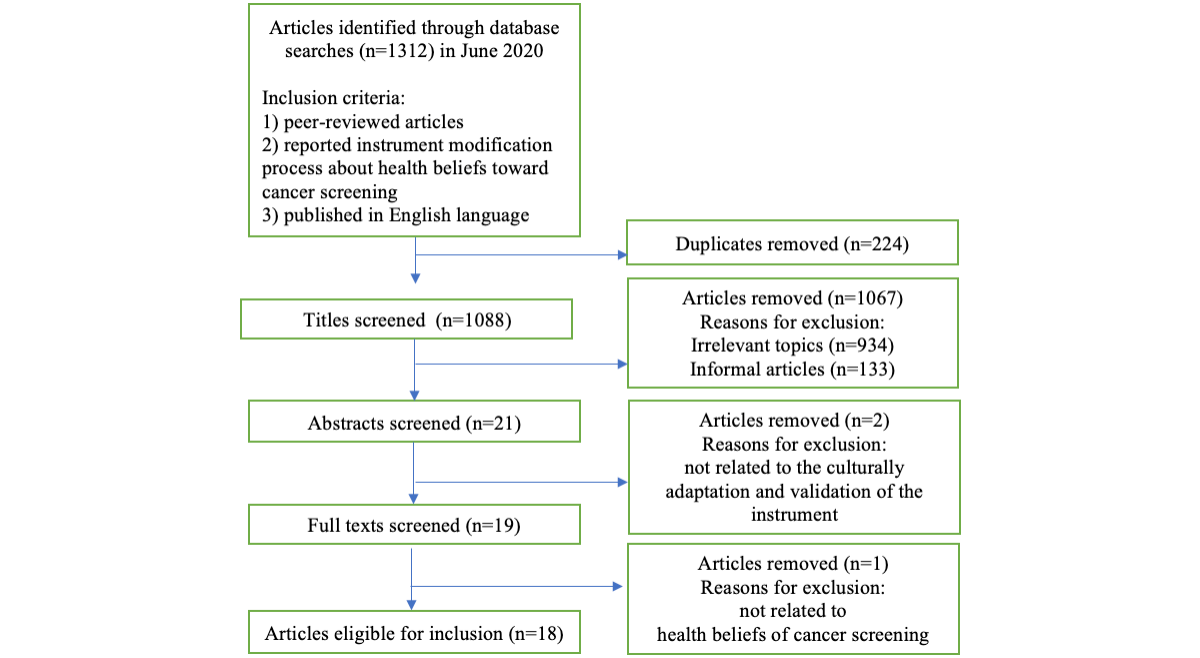
PRISMA flowchart documenting study selection process.

A total of 1312 articles were initially identified in the databases. After removing duplications, the titles and abstracts of the articles were assessed further, and 1293 articles were excluded in the process. Out of the 19 remaining articles screened, 1 article was further excluded because it focused on the cultural beliefs of cancer screening instead of health beliefs of cancer screening. In total, 18 studies met the inclusion criteria and were included in the study [22-39] (Figure 1).

### Study Characteristics

Among the 18 reviewed studies (Table 1), 5 were conducted in Turkey [22-26], 3 in Iran [27-29], 2 in the United States [30,31], and 1 each in Mexico [32], Indonesia [33], Malta [34], South Korea [35], Jordan [36], Malaysia [37], the city of Kaunas in Lithuania [38], and Cyprus [39]. The publication years of the studies ranged from 2001 to 2020. The sample size of the studies ranged from 15 to 656. Convenience sampling [22,25,28,32-35] and random sampling methods [23,27,29,36,37] were the most frequently used recruitment methods. Champion’s Health Belief Model Scale [14] was most commonly (17/18) adapted in the studies. The remaining study [30] created their scale by combining an adapted health belief scale from Menon et al [40] and a severity scale by Champion [41]. All studies were guided by the health belief model. Sixteen studies were about health beliefs of breast cancer screening and 2 studies were about health beliefs of colorectal and cervical cancer screening. All adapted instruments in the studies were proved to be valid and reliable through the validation process.

**Table 1:** Study characteristics for the included articles.

Study authors and citation	Modification strategies
Gozum and Aydin [22]	Rephrasing words in the items (S^a^)
Guvenc et al [23]	Adding items (C^b^)
Karayurt and Dramalı [24]	Rephrasing words in the items (S)
Secginli and Nahcivan [25]	Rephrasing words in the items (S)
Yilmaz and Sayin [26]	Rephrasing words in the items (S); deleting parts of the sentences in the items (C)
Hashemian et al [27]	Deleting parts of the sentences in the items (C); deleting items (C); adding items (C)
Kharameh et al [28]	Rephrasing words in the items (S); deleting items (C)
Taymoori and Berry [29]	Rephrasing words in the items (S); deleting parts of the sentences in the item (C); changing the subject of the items (C); changing the response options (S)
Lee and Lee [30]	Rephrasing words in the items (S); deleting parts of the sentences in the items (C); changing the response options (S)
Medina-Shepherd and Kleier [31]	Rephrasing words in the items (S)
Juárez-García et al [32]	Changing the response options (S); deleting items (C); adding items (C)
Dewi [33]	Rephrasing words in the items (S); deleting subscales (Cr^c^); adding subscales (Cr)
Marmarà et al [34]	Rephrasing words in the items (S); changing the subject of the items (C); deleting subscales (Cr); deleting items (C); adding subscales (Cr)
Lee et al [35]	Rephrasing words in the items (S)
Mikhail and Petro-Nustas [36]	Minor changes in the wording of the items (S); adding items (C)
Parsa et al [37]	Adding subscales (Cr)
Zelviene and Bogusevicius [38]	Rephrasing words in the items (S); deleting parts of the sentences in the items (C)
Tsangari and Petro-Nustas [39]	Minor changes in the wording of the items (S)

^a^S: semantic.

^b^C: content.

^c^CR: criterion.

### Data Evaluation and Extraction

The quality of the reviewed articles was evaluated by the first author and verified by the second author using Bowling’s checklist (Table 2). Data evaluation results showed the studies either had excellent or fair quality. All studies met 11 to 17 criteria on the checklist, although certain limitations existed (eg, generalizability of the findings to other populations: scales translated to one language cannot be used for another population speaking another language).

During the data evaluation process, the first author extracted relevant data from the reviewed articles and entered data into the Excel (Microsoft Corp) table of evidence, summarized correlated information into themes, and classified data into different categories. The second author checked the data and categories to ensure the findings were synthesized in a reliable way.

**Table 2:** Quantitative studies critical appraisal checklist [20].

	Criteria	Yes	No
1	Aims and objectives clearly stated	18	0
2	Hypothesis/research questions clearly specified	10	8
3	Dependent and independent variables clearly stated	3	15
4	Variables adequately operationalized	14	4
5	Design adequately described	12	6
6	Method appropriate	18	0
7	Instruments used tested for reliability and validity	18	0
8	Source of sample, inclusion/exclusion, response rates described	14	4
9	Statistical errors discussed	4	14
10	Ethical considerations described	13	5
11	Study was piloted	15	3
12	Statistically analysis appropriate	18	0
13	Results reported and clear	18	0
14	Results reported related to hypothesis and literature	18	0
15	Limitations reported	13	5
16	Conclusions do not go beyond limit of data and results	18	0
17	Findings able to be generalized	0	18
18	Implications discussed	18	0
19	Existing conflicts of interest with sponsor identified	0	18
20	Data available for scrutiny and reanalysis	0	18

### Modification Strategies

According to Flaherty’s cultural equivalence model, strategies used in the modification process were categorized by the equivalence type, especially semantic, content, and criterion equivalence (Table 3).

**Table 3:** Modification strategies used in the studies.

Equivalence type	Strategies
Semantic equivalence	Rephrasing items Rephrasing response options
Content equivalence	Changing subjects of items Changing wording of items Adding items Deleting items
Criterion equivalence	Adding subscales and items Deleting subscales and items

#### Semantic Equivalence

Semantic equivalence requires the meaning of each item in the adapted instrument to be similar to the meaning of the original item [19]. When aiming to achieve this type of cultural equivalence, rephrasing items and response options were frequently used in the reviewed studies.

##### Rephrasing Items

Upon reading expert and participant comments about the scales, the authors simplified and modified some wordings of items during the modification phase [28,33,36,39]. This strategy was frequently used in the reviewed studies (eg, some words in the items were replaced by other words, and medical terms were replaced by generally known terms).

To reach cultural accuracy of items, some words in the items were replaced by other words. Following one participant’s suggestion, in the study to adapt a Korean version of Champion’s Health Belief Model Scale, the word “hok” was changed into “meongwooli.” Even though both words meant lump or mass in Korean, the authors asserted the modification helped the women in the study to better understand the content of the instrument [35]. To accurately measure Turkish women’s health belief of breast cancer screening, the word “komik” (meaning funny) was changed into “tuhaf” (another expression of funny), and “gizlilik” (meaning privacy) was changed into “mahremiyet” (another expression of privacy) per expert suggestions. This change increased the consistency between the translated version and original version of the instrument [25]. The item “When I do breast screening examination, I feel good about myself” was changed to “I feel self-satisfied” as it was closer to the Iranian meaning than “feel good” [29].

Another strategy used to reach cross-cultural semantic equivalence was rephrasing medical terms to generally known terms. This strategy was used in the study conducted to measure health beliefs of colorectal cancer screening among Korean Americans [30]. The medical term “fecal occult blood test” was replaced with lay language “stool blood test,” as the medical term might be difficult to understand for participants not employed in a health-related field [30]. In the Turkish Health Belief Model Scale [26], the term “lump” was translated to “kitle” initially and changed to “sert yumrubeze,” a lay-language word that has a similar meaning to “kitle” and would be understood by Turkish women [26]. In the Maltese Health Belief Model Scale [34], “mammografija” was changed to “mammogram” and “nipil” was translated to “nipple” because “mammogram” and “nipple” are generally known terms with similar meanings to “mammografija” and “nipil” in Maltese. A similar strategy was used in the study conducted among Hispanic women [31]. The authors changed the original term of breast, “mama,” to “seno,” a word with similar meaning, since “seno” would be most understood in all Hispanic groups.

To avoid causing misunderstanding, confusion, and anxiety, some instrument items were changed by reversing direction of the meaning. This strategy was used in one study conducted with Maltese women [34]. The item “...will last for a short time” with reverse scoring was replaced with “...will last for a long time.” Then scores on the item did not have to be reversed.

##### Rephrasing Response Options

To increase clarity and achieve semantic equivalence, wordings of response options in the instruments may need to change. This strategy was used in 3 studies [29,30,32]. To adequately measure Mexican women’s health belief toward breast cancer screening, the response options were amended to 4=yes, 3=I think so, 2=I don’t think so, and 1=no, since the original response options were found to be problematic for the participants [32]. Also, instead of using the original response options (from “strongly disagree” to “strongly agree”), the Farsi Health Belief Model Scale used “not at all true” to “very true” for the perceived severity, susceptibility, benefits, and barriers subscales and “never” to “always” for the health motivation subscale, since most of the participants in the pretest phase reported problems with the format of response options [29]. Furthermore, the response option in the scale to measure Korean Americans’ health beliefs about colorectal cancer screening, “neutral” was changed to “so-so” per expert advice and suggestions in the published literature [30].

#### Content Equivalence

Content equivalence requires the content of each item in the adapted instrument to be relevant or appropriate to each cultural group or population under study [19]. This type of cross-cultural equivalence was usually achieved by changing subjects of items, changing wording of items, adding items, or deleting items.

##### Changing Subjects of Items

This strategy was used to achieve content equivalence by making the meanings of the items relevant or appropriate. It could avoid arousing fatalistic thoughts that were commonly present among minority populations. This strategy was used in the study conducted with Maltese women [34]. The item “My illness has serious...consequences” was replaced with “Breast cancer has serious...consequences.” The participants were asked to report their personal views about breast cancer instead of an illness personally affecting them [34]. Another strategy used in the study was using the third person pronoun instead of the first person pronoun to avoid arousing fatalistic thought. In some cultures, people believe that expressing an ominous event in the first person indicates that the event will occur [29]. In the Iranian Health Belief Model Scale [29], the first person pronoun in the item “If I developed breast cancer, I would not live longer than 5 years” was changed to the third person pronoun: “If someone developed breast cancer, she would not live longer than 5 years.” Similarly, changes were also made to another 3 items in the perceived severity scale in this study [29].

##### Changing Wording of Items

To modify items measuring the sizes of breast lumps in Champion’s Health Belief Model Scale (“I am able to find a breast lump which is the size of a quarter/dime/pea”), different items or coins familiar to the target population were used. In the Indonesian Health Belief Model Scale, “quarter” and “dime” were changed to “walnut” and “hazelnut,” respectively, because the sizes of a walnut and hazelnut are commonly known in Indonesia [33]. To find equal sizes to “quarter, dime, and pea,” the words were translated into “chickpea, hazelnut, and walnut” in the Turkish Health Belief Model Scale [25]. To represent dime and quarter in Iranian culture, the authors used “filbert” and “rather greater than filbert,” since “filbert” is commonly known in Iran culture [29]. In addition, the authors used different sizes of Turkish coins equalized to the sizes of the American quarter and dime, and the sizes of the original quarter and dime were given in centimeters in another study with Turkish women [26]. Similarly, wordings about the size of a palpable lump were also changed according to the sizes of currencies in Kaunas [38] and South Korea [35] in 2 other studies.

##### Adding Items

To increase the cultural sensitivity of the adapted instrument, thus increasing the scale’s content equivalence, entire items or parts of items were added to the adapted instrument.

Adding entire items to the modified scale was a common strategy used in the reviewed studies. In the Iranian Health Belief Model Scale, the item “I am more likely than the average woman to get breast cancer” was added to the adapted instrument because this item was maintained in the previous version of Champion’s scale and also because of the special features of the participants in the study (who had a family history of breast cancer) [27]. Furthermore, 4 items, (I don’t know where to go for mammography; I don’t have any problem with my breasts, I don’t need mammography; I do self-examination of the breasts, so there is no need for mammography; and I don’t have enough money for mammography) were added to the subscale of perceived barriers per participant discussion [27]. In addition, per expert suggestions, 2 items concerning awareness of the age and frequency at which mammograms should be undertaken were added to the self-efficacy subscale of the Mexican Health Belief Model Scale. Two items concerning myths were added to the barrier subscale. Two items concerning risk factors for cancer were added to the susceptibility subscale, and one item on drug use avoidance was added to the health motivation subscale [32].

To make the meaning of the items clear and easily understandable, additional words of explanation were added as part of some items. In the study conducted with Korean Americans, the explanation “not wanting to let other people know that you are doing the stool blood test or handling stool for the test” was added to the item “not having privacy would keep you from having a stool blood test,” since privacy could be interpreted as several different Korean words depending on the context [30]. Also, the item, “I have other problems more important than having a stool blood test,” was expanded to “Having a stool blood test is not the most urgent and important problem I have, which keeps me from doing it” because several participants reported they did not understand its meaning [30]. In the Turkish Health Belief Model Scale [26], the term “radyasyon” was explained as “radiation, x-ray/in other words radyasyon-rontgen” because some women in the target population would not know the word radiation since general understanding of the word was “x-ray.” Similarly, in the same study, “mammography” was expanded to “mammography” and “breast x-ray” in the modified scale because many women in Turkey do not know about early diagnostic methods for breast cancer, especially mammography [26]. Furthermore, in the translated Kaunas instrument, after pretesting with 10 women, 2 alternatives of the original word meaning “privacy,” solitude and severalty, were written with the original meaning next to the item “I don’t have enough privacy to do breast examination” [38].

##### Deleting Items

Deleting entire items or parts of items was a common strategy used in the studies to achieve content equivalence. Entire items in the reviewed studies were removed due to redundancy, irrelevance, or inaccuracy or a low content validity index at the item level. In one study, the item “I am too old to need a routine mammogram” was deleted because the Iranian women (n=200) in the study were younger (mean age 46.15 [SD 7.26] years, range 28 to 69 years) than the participants in the original Champion study (aged 50 years and older) [27]. Furthermore, the item “I don’t know how to go about getting a mammogram” was eliminated because the city was quite small [27]. Similarly, 2 items, “Breast cancer will last for a long time” and “I expect to have breast cancer for the rest of my life,” were removed in another study because the 2 items were found to confuse the Maltese participants and cause consistent heightened anxiety in responders [34]. Finally, the item “receiving a mammogram prior to breast screening” was deleted from a study to avoid overlap [34].

To reach cross-cultural content equivalence, parts of sentences in items from the original instruments were deleted. The terms “boyfriend” and “partner” in the item “Breast cancer would threaten a relationship with my boyfriend, husband, or partner” were deleted in 2 studies with Iranian women because sexual relationship outside the marriage is forbidden by Islamic rules and religious norm [29]. Furthermore, the word “blood” in the item describing “stool blood test” was deleted to emphasize that a stool sample was needed because some Korean American participants wrongly thought the test required blood to be taken [30].

#### Criterion Equivalence

Criterion equivalence requires the interpretation of an instrument’s relationship to established independent criteria for a certain event to be the same across cultures. This type of equivalence was usually achieved by adding subscales or items and deleting subscales.

##### Adding Subscales or Items

In the Maltese Health Belief Model Scale, the authors added subscales concerning the impact of sociodemographic and socioeconomic factors (eg, items about education level and income) on women’s breast screening behavior to acknowledge the contributions of those criteria to breast cancer screening [34]. A cues to action subscale (such as physician recommendations and family history), often omitted from empirical studies using Champion’s Health Belief Model Scale, was added because the authors thought cues to action were important criteria through which to examine the health belief of Maltese toward breast cancer screening [34]. In the Turkish Health Belief Model Scale, 4 items (cost, fatalism, preference for female health care professionals, and distance from the health center) thought to be appropriate to Turkish culture were added to the barrier subscale [23]. Furthermore, to test Jordan women’s fatalistic beliefs about breast cancer, the item “If I get sick with breast cancer, I believe this is my fate and practicing breast screening examination will not change my fate regardless of when the tumor is detected” was added to the barrier subscale [36].

##### Deleting Subscales or Items

In the reviewed studies, some subscales in the Health Belief Model Scale were deleted to increase cultural sensitivity. When the literature search indicated that most Maltese women perceived breast cancer to be a serious threat, the perceived severity scale was removed from the Maltese Health Belief Model Scale because the authors determined that perceived severity was not a criterion for examining the health belief of Maltese women toward breast cancer screening [34]. In addition, the item “people who perform mammograms are rude to women” was eliminated because participants believed that a sense of shame prevents them from receiving mammography instead of the issue of obscenity, and the statement lacked compatibility with Iranian culture [27].

#### Disagreement Solution Strategies

If a disagreement arose and panel members could not reach consensus on the translated items, solution strategies included consultation with experts, literature search, following the majority, and consultation with the author who developed the scales.

##### Consultation With Experts or Literature Search

This strategy was used in one study aiming to measure the health belief of Korean Americans toward colorectal cancer screening [30]. When the primary investigator and translation committee members encountered difficulty reaching a consensus on translation, they either sought guidance from an expert or literature published in both Korean and English to solve the dispute [30].

##### Following the Majority

This strategy was used in one study conducted with Hispanic women. The terms used for marital status aroused a disagreement over the comment from an expert panel member. The expert did not believe that every Hispanic would understand *estado civil* to mean marital status. However, a consensus was reached by the majority of the panel and the term *estado civil* was used in the translated instrument [31].

##### Consultation With the Author Who Developed the Scales

When meanings of the items in the original scale were not stated clearly, consulting with the author who developed the scales may provide clarification. In a study conducted with Korean Americans, some participants did not understand the meaning of the term “privacy” in the barrier items. Hence, the primary investigator consulted with the author who developed the barrier scale. The author clarified the meaning of privacy, and the item was rephrased accordingly [30].

## Discussion

### Summary

This study synthesized the modification strategies used in the instrument adaptation process to achieve cultural equivalence and provided solutions to the divergence in the instrument modification process. The instrument that measured health beliefs about cancer screening was used as an example in the study. To our knowledge, this is the first study to date investigating the modification strategies used in the adaptation process of instruments measuring health beliefs of cancer screening. The modification strategies to achieve cultural equivalence summarized in this study could help researchers gain insight into the instrument modification process.

To reach cross-cultural equivalence of the adapted instruments, modification is an essential step. According to Medina-Shepherd and Kleier [31], studies using cross-cultural instruments without the process of modification may have problems with validity. To make the instrument culturally appropriate, researchers must use words that are preferred and commonly used by the target population. If appropriate attention is not given to word choice, the instrument may be meaningless to participants from the target population, and accurate responses might not be obtained [42]. Therefore, changes and adaptation of the items in the source language may be necessary to achieve cultural equivalence in the target language.

According to the literature, the strategies used in the adaptation process generally included 3 types of modifications—changing, deleting, and adding—and 2 levels—scale level and item level, which consisted of modification of the question statement and response options).

First, rephrasing items or response options was a basic strategy in instrument modification to achieve semantic equivalence [28,33,36,39]. Following expert and participant suggestions, changes to wording could be made on specific items and response options. In addition, medical terms may need to be rephrased to generally known terms to enhance understanding, and confusing items may need to change their directions of meaning. In the instrument modification process, clarity is an important criterion that should be considered. If a statement in the modified instrument is not clearly understood by the participants or causes confusion, the wording should be further changed or modified to reach accuracy at the item level [35]. Medical terms not well known in the lay population need to be modified to give participants more insight into the instrument questions [30]. Replacing the medical term with a generally known term [34] or adding an explanation to the medical term can assure an easier understanding. Changing the direction of the meaning can lessen confusion caused by the statement as well [34]. Although changing the wording of an instrument is easily achieved, cross-cultural researchers still need to use this strategy with careful consideration. Consultation with experts and participants from the target population is still the most important step to validate the modification.

Second, changing subjects of items, changing wordings, and adding or deleting items could help the adapted instrument achieve content equivalence with the original items. Some item subjects may be not appropriate in the instrument due to fatalistic thoughts of participants, and the subjects may need to be changed. Adding relevant items and deleting irrelevant statements were also important strategies to reach content equivalence. In the literature reviewed in this study, items tended to be added to increase cultural sensitivity and clarity and deleted to decrease redundancy, irrelevance, or inaccuracy or increase the content validity index at the item level. In the instrument modification process, items in the original scale suitable to the initial cultural context may not be suitable to the other cultural context. Selecting relevant items and deleting irrelevant items could lessen confusion and make the scale more meaningful [32]. Expanding the incomplete statement by adding an explanation or instruction for answering the question could help participants fully and clearly answer the question [30], increasing the response rate for each item. However, adding or deleting items should be carefully considered since the modification may impact the instrument’s reliability and validity. Pilot testing of modified instrument’s validities (content, construct, predictive, and face validity) and reliabilities (internal consistency and test-retest reliability and item-total subscale correlations) is necessary before launching the modified instrument into formal use.

In addition, the strategies of adding and deleting subscales and items were often used to achieve cross-cultural criterion equivalence. In the literature reviewed in this study, the specific reason for adding and deleting subscales and items was to increase cultural sensitivity and clarity. This strategy should be used with careful consideration. Unless supported by a comprehensive literature review or updated theoretical framework that reflects a changed base of the instrument, adding or deleting subscales and items could significantly impact the validity of the adapted instrument.

Furthermore, disagreement solution strategies for the modification process included consultation with experts or literature search, following the majority, and consultation with the author who developed the scales. Using an appropriate solution strategy to solve a disagreement arising in the modification process can clarify the vague meanings of items and further increase the validity of the items. During the modification process, it is best to have a research team with bilingual professionals who are familiar with the cultures for which the instrument was originally developed and to which it will later be adapted. If it is possible, the primary investigators for the instrument modification should be the ones who are bilingual, bicultural, and familiar with the concepts measured in the instrument. This could facilitate the instrument modification process and help meet challenges that emerge during the process.

### Limitation

This systematic review has some limitations. First, we used a narrative rather than a meta-analysis method to summarize data. As such, our findings cannot be used to recommend the optimal strategies for modifying instruments used in the cross-cultural research. Second, we reviewed only articles written in English, which may have biased the data and restricted our findings. Limiting the review to English language articles may introduce a language bias and lead to erroneous conclusions [43]. However, since 92.50% of scientific literature is written in English [44], the language bias may have little impact on this study. Third, modification strategies synthesized in this study may not be able to reflect other factors impacting the modification process. Factors such as personal experience and expertise of the researcher, translator, or interpreter; educational level and health literacy of the target population; and cultural integration and assimilation levels between populations should also be considered in the modification process.

### Future Direction of Research

Instrument modification is an important part of cross-cultural research. Adapting an instrument developed for another culture to be used in the target population can save time, add value to the original instrument, and promote science achievements to circulate around the world. With the development of science, factors impacting the cross-culture instrument modification change accordingly. For example, instruments used for online and offline cancer screening (eg, paper version, telephone assessment) may differ in wording, which may impact the technical equivalence of the instrument. A systematic review of the factors that may impact the instrument modification process in the new stage of science is necessary and can help cross-cultural researchers gain a comprehensive understanding of the modification process to achieve cultural equivalency. In addition, research to update the definition of cross-cultural equivalence and a clear gold standard checklist to evaluate cultural equivalence for the instrument modification should be established for cross-cultural researchers, since Flaherty’s approach was introduced several decades ago [19]. This could help to examine the cultural equivalence of the modified instrument to the original instrument and further increase the modified instrument’s validity and reliability.

### Conclusions

Instrument modification is a necessary process in cross-cultural instrument adaptation. This study summarized the modification strategies used to culturally adapt instruments measuring health beliefs of cancer screening to achieved cross-cultural equivalence. It can potentially give cross-cultural researchers more insight into the modification strategies and a better understanding the modification process in the cross-cultural instrument adaptation. More research needs to be done to help researchers better modify cross-cultural instruments and develop a checklist to achieve cross-cultural equivalence.

## Appendix

Multimedia Appendix 1 PRISMA checklist.

## Abbreviations

PRISMA: Preferred Reporting Items for Systematic Reviews and Meta-analyses
